# Membrane-Mediated Interactions Between Protein Inclusions

**DOI:** 10.3389/fmolb.2021.811711

**Published:** 2021-12-22

**Authors:** Jie Gao, Ruihan Hou, Long Li, Jinglei Hu

**Affiliations:** ^1^ Kuang Yaming Honors School, Nanjing University, Nanjing, China; ^2^ State Key Laboratory of Nonlinear Mechanics (LNM) and Beijing Key Laboratory of Engineered Construction and Mechanobiology, Institute of Mechanics, Chinese Academy of Sciences, Beijing, China

**Keywords:** lipid membrane, protein inclusions, membrane-mediated interactions, nanoparticles, membrane elasticity, molecular dynamics, Monte Carlo

## Abstract

Integral or peripheral membrane proteins, or protein oligomers often get close to each other on cell membranes and carry out biological tasks in a collective manner. In addition to electrostatic and van der Waals interactions, those proteins also experience membrane-mediated interactions, which may be necessary for their functionality. The membrane-mediated interactions originate from perturbation of lipid membranes by the presence of protein inclusions, and have been the subject of intensive research in membrane biophysics. Here we review both theoretical and numerical studies of such interactions for membrane proteins and for nanoparticles bound to lipid membranes.

## 1 Introduction

The cell membrane is a fluid bilayer made up of lipids and proteins. The proteins are associated with the bilayer either *via* insertion of hydrophobic domains into one or two monolayers, or *via* covalent linkage or reversible adsorption to the lipids (Alberts et al., 2015). These proteins perform various biological tasks often in a collective manner. For example, two gramicidin molecules (linear peptides) each embedded in one leaflet of the bilayer dimerize in a head-to-head fashion to form a membrane-spanning ion channel with the dimerization rate dependent on the bilayer tension. In clathrin-mediated endocytosis, a multitude of proteins assemble on the cell membrane to form the clatherin-coated endocytic vesicle of size around 100 nm (Kaksonen and Roux, 2018). In addition to electrostatic and van der Waals interactions, those proteins that are spatially close on the membrane also experience membrane-mediated interactions, which may play an important part in membrane-associated processes.

In this review we present an overview of both theoretical and numerical studies on membrane-mediated interactions between protein inclusions. Protein inclusion is used here as a general term that refers to integral and peripheral membrane proteins or protein oligomers, and nanosized colloidal particles adhering to lipid membranes. The direct protein-lipid interactions lead to indirect, membrane-mediated interactions between the proteins. These interactions consist of short- and long-range parts. The former arises from local perturbation of bilayer structure by the protein inclusions, which decays over a length of around the protein size or bilayer thickness. The latter is ascribed to both modification of membrane fluctuations and perturbation of membrane equilibrium shape due to the presence of protein inclusions. The long-range interaction is treated on length scales larger than the protein size or membrane thickness. In Section 2, we discuss theoretical investigations that have shed lights on how different physical factors contribute to the short- and long-range interactions, and briefly describe the continuum approaches for deriving the interactions based on bilayer elasticity models. In Section 3, we focus on numerical studies that have quantified the short- or long-range interactions, with emphasis on different coarse-grained models for the fluid membranes. We also make qualitative comparison between numerical results and theoretical predictions. In this mini-review we do not discuss the experimental studies. Interested readers are referred to Refs (Bitbol et al., 2018; Idema and Kraft, 2019).

## 2 Theory

### 2.1 Short-Range Interaction

Insertion of protein molecules into a lipid bilayer perturbs the packing of nearby lipid chains, since the protein-lipid and lipid-lipid interactions are generically different. Such local perturbation leads to entropy loss of the lipid chains and induces a short-range interaction between the proteins. By defining an order parameter for lipid chain orientation, Marčelja (1976) constructed a mean-field Hamiltonian for a model system of two hexagonal proteins embedded in a flat lipid bilayer and predicted a pure attraction between the proteins. By taking the same assumption that fluctuations of lipid orientation are suppressed in the protein vicinity, Schröder (1977) derived an expression for the attraction. The attraction arising from lipid orientational entropy was first verified by coarse-grained Monte Carlo simulations (Sintes and Baumgärtner, 1997), as will be discussed in Section 3.1. May and Ben-Shaul (2000) applied detailed molecular chain packing theory to calculate the interaction between two protein walls in a bilayer and found that the interaction is attractive at small separations and repulsive at intermediate separations. This interaction starts to level off at separation around the hydrophobic thickness of the proteins or bilayer. Nonmonotonic lipid-mediated interaction potentials between protein inclusions were also reported by Lagüe et al. (2001) based on integral equation theory for liquids.

Protein inclusions generally exhibit different hydrophobic thickness from the embedding lipid bilayer. Lipid chains surrounding the inclusions will stretch or compress in order to avoid or alleviate the exposure of hydrophobic regions of the proteins or lipids. This deformation represents another source of free energy cost that contributes to the bilayer-mediated short-range interaction. Owicki and McConnell (1979) presented a Landau-de Gennes free energy to account for lipid chain deformation and bilayer area change caused by hydrophobically mismatched inclusions, and obtained a short-range attraction between such two inclusions. Huang (1986) formulated a continuum theory to describe the bilayer deformations around a rigid inclusion by considering the free energies associated with monolayer bending, lipid chain compression and surface tension, and reported a nonmonotonic bilayer thickness profile around single inclusions. Dan et al. (1993) and Aranda-Espinoza et al. (1996) adopted this continuum theory to calculate the membrane-mediated interaction between two cylindrical inclusions with hydrophobic mismatch. The interaction looks qualitatively similar, in the case of vanishing spontaneous curvature of the monolayers, to that obtained in Ref (May and Ben-Shaul, 2000), and has a range of about two to three times of the bilayer thickness.

**FIGURE 1:**
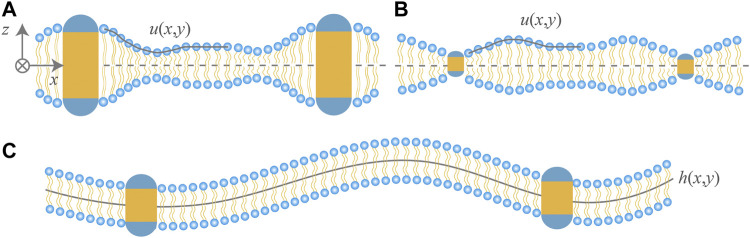
Cartoon of lipid bilayers containing two rigid cylindrical proteins. Hydrophilic regions of the proteins are shown in blue and hydrophobic in yellow. Hydrophobic thickness of the proteins in **(A**,**B)** is, respectively, greater and less than that of the bilayer in the unperturbed state. For up-down symmetric bilayers, the thickness deformations due to hydrophobic mismatch are described by the profile *u*(*x*, *y*) of the upper monolayer. The protein inclusions in **(C)** have the same hydrophobic thickness as the bilayer and modify the shape fluctuations of the bilayer as can be characterized by the midplane profile *h*(*x*, *y*). The bilayer midplane in **(A**,**B)** is drawn to be horizontal for simplicity, since thickness deformations decouple from midplane deformations.

The continuum approach (Huang, 1986; Dan et al., 1993; Aranda-Espinoza et al., 1996) based on membrane elasticity has been widely used to investigate the role of membrane-mediated interactions in such membrane protein processes as formation of gramicidin ion channels (Huang, 1986; Bitbol et al., 2012; Bories et al., 2018), cooperative gating of mechanosensitive channel of large conductance (Ursell et al., 2007; Haselwandter and Phillips, 2013; Kahraman et al., 2016a) and assembly of chemoreceptor trimers (Haselwandter and Wingreen, 2014). Extensions of the membrane elasticity model have been made by including lipid tilt (Fournier, 1999; Bohinc et al., 2003), Gaussian curvature (Brannigan and Brown, 2007), gradient of bilayer thickness (Bitbol et al., 2012), and asymmetry in two monolayers due to noncylindrical shape of the inclusions (Argudo et al., 2017). We briefly describe this approach for a up-down symmetric and single-component lipid bilayer. As shown in Figures 1A,B, the thickness deformations of the lipid bilayer around a hydrophobically mismatched protein inclusion are characterized by the relative displacement *u*(*x*, *y*) of the upper monolayer with respect to the horizontal midplane. By a Taylor-expansion around the unperturbed flat state of the bilayer with thickness 2*u*_0_ and area per lipid Σ_0_, the monolayer free energy can be expressed in terms of *u*, gradient of *u* (i.e., ∇*u*), mean curvature *H* ≈ ∇^2^ *u*/2, and Gaussian curvature *K* ≈ det(∇∇*u*). The free energy of monolayer compression or stretching per projected area is $f_{\text{c}}=K_{\text{A}}{\left(u/u_{0}\right)}^{2}/4$ with *K*_A_ the bilayer’s area compression modulus. The corresponding surface-tension term is *f*_t_ = *σ*[*u*/*u*_0_ + (∇*u*)^2^/2]/2 (Huang, 1986; Haselwandter and Phillips, 2013; Kahraman et al., 2016b) with *σ* the bilayer tension. The bending energy density assumes $f_{\text{b}}=\left[\kappa {\left(\nabla^{2}u\right)}^{2}/2+\kappa c_{0}\nabla^{2}u+\kappa \left(c_{0}-c_{0}^{\prime }\Sigma_{0}\right)\left(u/u_{0}\right)\nabla^{2}u+\bar{\kappa }\;\text{det}\left(\nabla \nabla u\right)\right]/2$ (Dan et al., 1993; Aranda-Espinoza et al., 1996; Brannigan and Brown, 2007), where *κ* is the bilayer bending rigidity, *c*_0_ the monolayer spontaneous curvature, $c_{0}^{\prime }={\left(\partial c_{0}/\partial \Sigma \right)}_{\Sigma_{0}}$ the change of *c*_0_ due to lipid area variation, and $\bar{\kappa }$ the bilayer Gaussian modulus. The total free energy of the perturbed monolayer per inclusion is then given by the functional $F\left[u\right]=\int \int \text{d}x\text{d}y\left(f_{\text{c}}+f_{\text{t}}+f_{\text{b}}\right)$ . Minimization of F[u] under appropriate boundary conditions (Nielsen et al., 1998; Nielsen and Andersen, 2000; Brannigan and Brown, 2006) determines the bilayer deformations around the inclusions.

### 2.2 Long-Range Interaction

There exist two types of long-range interactions between protein inclusions mediated by the embedding fluid membranes: fluctuation-induced interactions and curvature-induced elastic interactions. As the name suggests, they originate, respectively, from modification of membrane fluctuations and from perturbation of the equilibrium membrane shape by the presence of protein inclusions. In theoretical considerations, the fluid membrane, on length scales larger than its thickness, is coarse-grained into a two-dimensional (2D) elastic surface governed by the Helfrich Hamiltonian (Helfrich, 1973) $H_{\text{el}}=\int \left[\kappa {\left(2H-c_{0}\right)}^{2}/2+\bar{\kappa }K+\sigma \right]\text{d}A$ , where *H* is the membrane’s mean curvature, *K* the Gaussian curvature, *c*_0_ the spontaneous curvature due to bilayer asymmetry, *κ* the bending rigidity, $\bar{\kappa }$ the Gaussian modulus, and *σ* the lateral tension conjugate to the membrane area. Typical values for the physical properties of synthetic and biological membranes are *κ* ∼ 10*k*_B_ *T* ≈ 4 × 10^−20^J, $-\kappa \leq \bar{\kappa }\leq -0.7\kappa$ (Deserno, 2015), and *σ* ∼ 1 *μ*N/m (Simson et al., 1998). $\ell \equiv \sqrt{\kappa /\sigma }∼200\;\text{nm}$ is the characteristic length scale over which the surface tension dominates over the bending energy. A membrane surface that exhibits small deformations is parameterized by using a displacement field with respect to a planar, spherical, or cylindrical reference surface; *see* Figure 1C. The Helfrich Hamiltonian is then a function of the displacement field. Proteins that are embedded in or attached to the membrane are treated by applying boundary conditions to the displacement field or *via* extra energy terms that enter the Hamiltonian of the system. The fluctuation-induced interaction can be estimated from cumulant expansion, whereas the curvature-induced elastic interaction is often derived by finding the minimum-energy shape of the membrane under appropriate boundary conditions and additional requirements that the inclusions are in mechanical equilibrium, i.e., force- and torque-free. In all the following, we will use the notations $F_{\text{fl}}$ and $F_{\text{el}}$ in energy units to distinguish the two types of long-range interactions, and cite the leading-order expressions for $F_{\text{fl}}$ and $F_{\text{el}}$ as derived in literature unless specified otherwise.

#### 2.2.1 Protein Inclusions in Quasiplanar Membranes

Theoretical account of membrane-mediated long-range interactions between protein inclusions was pioneered by Goulian et al. (1993), who considered two proteins of circular cross section embedded in a tensionless fluid membrane, and discovered that thermal fluctuations of the membrane induce a long-range interaction between the inclusions. This fluctuation-induced interaction $F_{\text{fl}}$ decays as 1/*R* ^4^ for interprotein separation *R* much larger than the protein radius or membrane thickness *a*, given that the bending rigidity *κ*_p_ and Gaussian modulus ${\bar{\kappa }}_{\text{p}}$ of the proteins differ from those of the membrane. More specifically, for infinitely rigid proteins ( $\kappa_{\text{p}}=-{\bar{\kappa }}_{\text{p}}=\infty$ ), $F_{\text{fl}}\approx -6k_{\text{B}}T{\left(a/R\right)}^{4}$ (Goulian et al., 1993; Park and Lubensky, 1996) is purely attractive with magnitude set by the thermal energy *k*_B_ *T* and independent of the membrane rigidities *κ* or $\bar{\kappa }$ . Very similarly, $F_{\text{fl}}\approx -k_{\text{B}}Tcos^{2}\left(2\theta_{1}+2\theta_{2}\right)/128{\left(\sqrt{l_{1}l_{2}}/R\right)}^{4}$ when the rigid inclusions are two thin rods of length *l*_*i*_ and orientational angle *θ*_*i*_ (*i* = 1, 2) relative to the vector joining their centers (Golestanian et al., 1996). The membrane-mediated, fluctuation-induced attraction is of entropic origin and arises from the fact that the number of allowed modes in the membrane is suppressed by the presence of rigid inclusions. Helfrich and Weikl (2001) indeed rederived the expression $F_{\text{fl}}\approx -6k_{\text{B}}T{\left(a/R\right)}^{4}$ for two rigid discoidal inclusions from fluctuation mode entropies of the membrane. For soft protein inclusions that have rigidities close to the membrane, i.e., *κ*_p_ = *κ* + Δ*κ* and ${\bar{\kappa }}_{\text{p}}=\bar{\kappa }+\Delta \bar{\kappa }$ with |Δ*κ*/*κ*|≪ 1 and $|\Delta \bar{\kappa }/\bar{\kappa }|\ll 1$ , $F_{\text{fl}}\approx \Delta \kappa \Delta \bar{\kappa }/\left(2\kappa^{2}\right)k_{\text{B}}T{\left(a/R\right)}^{4}$ (Goulian et al., 1993; Park and Lubensky, 1996), where the relative sign of Δ*κ* and $\Delta \bar{\kappa }$ dictates whether the interaction is attractive or repulsive. When $\Delta \kappa \Delta \bar{\kappa }<0$ , the soft inclusions experience a fluctuation-induced attraction, consistent with the limiting case of infinitely rigid proteins. Lin et al. (2011) developed a method to deal with two discs of arbitrary rigidities in a membrane under tension, and obtained in the *bending-dominated* regime ( $a\ll R\ll \ell \equiv \sqrt{\kappa /\sigma }$ ) the fluctuation-induced interaction $F_{\text{fl}}\approx -k_{\text{B}}T{\left(a/R\right)}^{4}f\left(\kappa ,\Delta \kappa ,\Delta \bar{\kappa }\right)$ with the dimensionless coefficient $f\left(\kappa ,\Delta \kappa ,\Delta \bar{\kappa }\right)=2\Delta \bar{\kappa }\left(3\Delta {\bar{\kappa }}^{2}+6\Delta \kappa \Delta \bar{\kappa }-8\kappa \Delta \kappa \right)/\left[{\left(4\kappa -\Delta \bar{\kappa }\right)}^{2}\left(2\kappa +2\Delta \kappa +\Delta \bar{\kappa }\right)\right]$ . This formula for $F_{\text{fl}}$ applies to protein inclusions of circular cross section, and successfully reproduces the previous two expressions obtained in the case of tensionless membranes (*σ* = 0), since $f\left(\kappa ,\Delta \kappa ,\Delta \bar{\kappa }\right)=6$ in the rigid-inclusion limit ( $\Delta \kappa =-\Delta \bar{\kappa }=\infty$ ), and $f\left(\kappa ,\Delta \kappa ,\Delta \bar{\kappa }\right)\approx -\Delta \kappa \Delta \bar{\kappa }/\left(2\kappa^{2}\right)$ for soft inclusions with |Δ*κ*/*κ*|≪ 1 and $|\Delta \bar{\kappa }/\bar{\kappa }|\ll 1$ . In the *tension-dominated* regime (*ℓ* ≪ *a* ≪ *R*), $F_{\text{fl}}\approx -9k_{\text{B}}T{\left(a/R\right)}^{8}$ for rigid proteins as also obtained in Ref (Yolcu et al., 2011) by using a different approach based on effective field theory, whereas $F_{\text{fl}}\approx -k_{\text{B}}T{\left[\Delta \bar{\kappa }/\left(a^{2}\sigma \right)\right]}^{2}{\left(a/R\right)}^{8}$ for soft inclusions. This 1/*R* ^8^ attraction is different from the 1/*R* ^4^ attraction for two thin rods embedded in a tension-controlled fluctuating film without curvature-energy term (Golestanian et al., 1996), possibly due to the different shapes. It is remarkable that in both the bending- and tension-dominated regimes, the fluctuation-induced interaction vanishes at $\Delta \bar{\kappa }=0$ , namely, for protein inclusions of the same Gaussian modulus as the membrane. The importance of Gaussian curvature to the fluctuation-induced interactions has been already appreciated in the simulation study of aggregation of rigid membrane inclusions (Weikl, 2001), as will be discussed later in Section 3.

**FIGURE 2:**
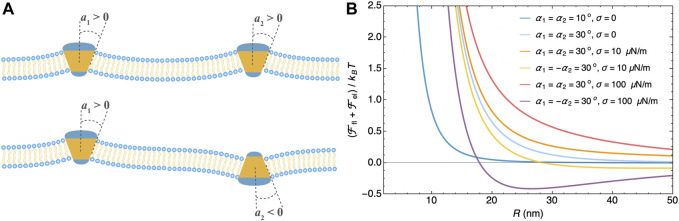
Membrane-mediated interaction between two conical inclusions. **(A)** Cartoon of membranes with two conical inclusions that have contact angles *α*_1_ and *α*_2_. **(B)** Membrane-mediated interaction as a function of the inter-inclusion distance *R* at different contact angles and membrane tensions calculated from $F_{\text{fl}}\approx -6k_{\text{B}}T{\left(a/R\right)}^{4}$ and $F_{\text{el}}\approx 2\pi \kappa \alpha_{1}\alpha_{2}{\left(a/\ell \right)}^{2}K_{0}\left(R/\ell \right)+\pi \kappa \left(\alpha_{1}^{2}+\alpha_{2}^{2}\right){\left(a/\ell \right)}^{4}K_{2}^{2}\left(R/\ell \right)$ (Weikl et al., 1998) with *a* = 5 nm and membrane bending rigidity *κ* = 25 *k*_B_ *T*. These two equations are explained in the main text.

Protein inclusions of shapes that break the bilayer’s up-down symmetry, e.g., cone shape, bend the membrane. Perturbation of the equilibrium membrane shape induces long-range interactions between such inclusions and has been taken into account by imposing either a contact angle (Goulian et al., 1993; Park and Lubensky, 1996; Weikl et al., 1998; Kim et al., 1998) or a curvature tensor (Dommersnes and Fournier, 1999a; Yolcu and Deserno, 2012) at the protein-membrane boundary. For rigid conical inclusions in a tensionless membrane (Goulian et al., 1993; Park and Lubensky, 1996; Weikl et al., 1998; Kim et al., 1998; Dommersnes and Fournier, 1999a,b; Yolcu and Deserno, 2012), the curvature-induced elastic interaction $F_{\text{el}}\approx 4\pi \kappa \left(\alpha_{1}^{2}+\alpha_{2}^{2}\right){\left(a/R\right)}^{4}$ is repulsive and depends on the contact angle *α*_*i*=1,2_ defined by the protein’s axis of rotational symmetry and the normal of the membrane at the boundary as shown in Figure 2A. For rigid conical inclusions in a membrane under tension, the elastic interaction obtained at *a* < *ℓ* takes the form $F_{\text{el}}\approx 2\pi \kappa \alpha_{1}\alpha_{2}{\left(a/\ell \right)}^{2}K_{0}\left(R/\ell \right)+\pi \kappa \left(\alpha_{1}^{2}+\alpha_{2}^{2}\right){\left(a/\ell \right)}^{4}K_{2}^{2}\left(R/\ell \right)$ (Weikl et al., 1998) with *K*_*n*=0,2_(*x*) the modified Bessel function of the second kind. This expression recovers the previous one for the case of vanishing membrane tension by approaching the limit *ℓ* → *∞*, and implies that the presence of membrane tension can render the elastic interaction between two conical inclusions of opposite orientations (*α*_1_ *α*_2_ < 0) attractive at separation *R* > *R* ^∗^. *R* ^∗^ is the separation for the minimum of $F_{\text{el}}$ and assumes *R* ^∗^ ≈ 6.6*a* for a reasonable choice of parameters *a*/*ℓ* = 0.1 and *α*_1_ = − *α*_2_ ≠ 0. *See* Figure 2B for the elastic interaction between two conical inclusions.

Shape anisotropy of the protein inclusions (Park and Lubensky, 1996; Dommersnes and Fournier, 1999a, 2002; Chou et al., 2001; Yolcu and Deserno, 2012), external torques on the inclusions (Dommersnes and Fournier, 1999b), and forces exerted on the membranes by the inclusions (Evans et al., 2003) can alter the membrane-mediated interactions. Park and Lubensky (1996) characterized protein inclusions of noncircular cross section by using symmetric-traceless tensor order parameters, and found that *1*) the fluctuation-induced interaction $F_{\text{fl}}$ is anisotropic and can be attractive or repulsive depending on the relative orientations of the inclusions to their center-to-center vector and that *2*) up-down asymmetry of the inclusion shape changes the distance dependence of $F_{\text{fl}}$ from 1/*R* ^4^ to 1/*R* ^2^. Chou et al. (2001) reported that the elastic interaction $F_{\text{el}}$ between two inclusions of elliptic cross section averaged over their orientations changes from repulsive to attractive with increasing ellipticity. Dommersnes and Fournier (1999a, 2002), Yolcu and Deserno (2012), and Noguchi and Fournier (2017) showed that, for wedge-shaped, saddle-like, or arc-shaped protein inclusions that impose anisotropic curvature on a tensionless membrane, the leading term of the curvature-induced elastic interaction $F_{\text{el}}$ is of 1/*R* ^2^-order and can be attractive or repulsive depending on the imposed curvatures and orientations of the inclusions. Dommersnes and Fournier (1999b) investigated the membrane-mediated interactions between two protein inclusions with orientations restricted by external torques, and revealed that the presence of external torques strongly increases the range of both the fluctuation-induced attraction $F_{\text{fl}}$ and curvature-induced repulsion $F_{\text{el}}$ . Specifically, $F_{\text{fl}}$ is found to be a function of ln *R*, whereas $F_{\text{el}}$ decays as 1/*R* ^2^ for the two inclusions with parallel orientations, or approximately as   ln(1/*R*) otherwise. Evans et al. (2003) calculated the elastic interaction between two cylindrical inclusions that apply normal forces to the membrane under tension and cause a variation in the membrane profile, and obtained the relation $F_{\text{el}}\propto K_{0}\left(R/\ell \right)/\left(2\pi \sigma \right)$ with the omitted coefficient of proportionality measuring the strength of membrane-inclusion coupling due to the forces. This curvature-induced elastic interaction is repulsive and decays slower than 1/*R* ^4^.

In addition to the aforementioned two-body interactions, there exist many-body interactions between the protein inclusions mediated by the membrane, which can not be simply accounted for by a sum of two-body interactions. Dommersnes and Fournier (2002) showed that the elastic interaction between three identical rigid conical inclusions in an equilateral-triangle arrangement is $F_{\text{el}}\approx 12\pi \kappa \alpha^{2}{\left(a/R\right)}^{4}$ , 50% less than the estimation of 24*πκα* ^2^(*a*/*R*)^4^ by assuming pairwise additivity. Yolcu and Deserno (2012) obtained general expressions for three- and four-body interactions between rigid conical inclusions in the case of vanishing membrane tension. For instance, the three-body fluctuation-induced interaction $F_{\text{fl}}\approx 4k_{\text{B}}T{\left(a_{1}a_{2}a_{3}\right)}^{2}/{\left(R_{12}R_{23}R_{31}\right)}^{2}\sum_{\left(i,j,k\right)}cos\left(2\vartheta_{ijk}-2\vartheta_{jki}\right)$ , where *a*_*i*_ is the cross-sectional radius of protein *i*, $R_{ij}\equiv |{\vec{R}}_{ij}|$ the interprotein distance, *ϑ*_*ijk*_ the angle between the distance vectors ${\vec{R}}_{ij}$ and ${\vec{R}}_{jk}$ , and the summation is over three cyclic permutations (*i*, *j*, *k*) = (1, 2, 3), (2, 3, 1), (3, 1, 2); the three-body elastic interaction $F_{\text{el}}\approx 8\pi \kappa \sum_{\left(i,j,k\right)}a_{i}^{2}a_{j}a_{k}\alpha_{j}\alpha_{k}/{\left(R_{ij}R_{ki}\right)}^{2}cos2\vartheta_{kij}$ with *α*_*i*_ the contact angle for protein *i*. The angular part in the above expression for $F_{\text{el}}$ suggests that this interaction can be attractive and might stabilize aggregates of protein inclusions. Dommersnes and Fournier (1999a) derived the three-body elastic interaction for inclusions that induce anisotropic curvatures onto a tensionless membrane. Weitz and Destainville (2013) followed the approach in Ref (Dommersnes and Fournier, 1999a) to calculate the three-body interactions for rigid conical inclusions in the case of nonvanishing membrane tension. These studies demonstrated that the three- and four-body interactions are of the same order of magnitude as the two-body counterparts and depend on the spatial arrangement of the proteins. Nevertheless, the two-body interactions may serve as good approximations at low concentrations of protein inclusions.

#### 2.2.2 Non-transmembrane Proteins Attached to Quasiplanar Membranes

In biological or biomimetic systems, the proteins can also be attached to lipid membranes in such a way that their center-of-mass positions are not at the center of the membrane bilayer. Park and Lubensky (1996) calculated the curvature-induced elastic interactions between non-transmembrane proteins that are bound to a membrane and have preferred center-of-mass positions off the bilayer midplane, and found that the three- and four-body interactions have a similar magnitude as the two-body interaction, and that all these interactions decrease with the distance as 1/*R* ^4^; *see* Eqs. 5.11–5.13 in Ref (Park and Lubensky, 1996). Weikl (Weikl, 2003) considered the elastic interactions between two infinitely long and parallel cylinders of radius *a* adhering to a membrane under tension. For cylinders bound to the same membrane side, $F_{\text{el}}\approx -{\left(\kappa +2a^{2}u_{\text{ad}}\right)}^{2}/\left(4\kappa a/\ell \right)\left\{1+tanh\left[R/\left(2\ell \right)\right]\right\}$ is repulsive; for cylinders adhering at opposite membrane sides, $F_{\text{el}}\approx -{\left(\kappa +2a^{2}u_{\text{ad}}\right)}^{2}/\left(4\kappa a/\ell \right)\left\{1+cosh\left[R/\left(2\ell \right)\right]\right\}$ becomes attractive. *u*_ad_ < 0 is the adhesion energy per area. Müller et al. (2005) reproduced these results by calculating the membrane-mediated elastic forces *via* line integral of stress tensor. Mkrtchyan et al. (2010) revisited the elastic interactions between two membrane-bound cylinders, and also observed attractions between cylinders strongly adhering to the opposite side of the membrane from numerical calculations.

#### 2.2.3 Protein Inclusions in Vesicular or Tubular Membranes

The previously discussed studies focused on quasiplanar membranes with small deviations from the reference flat state. For proteins embedded in vesicle or tubular membranes, the membrane size that characterizes the background curvature of the shape appears to be a relevant length scale. Dommersnes et al. (1998) calculated the elastic interaction between two identical conical inclusions in a spherical vesicle with fixed surface area, and found the interaction is always repulsive and proportional to the square of the contact angle. They recovered, at small separations, the 1/*R* ^4^ repulsion as reported in Ref (Goulian et al., 1993), and observed a much stronger repulsion that decays as 1/*R* ^0.33^ for separations larger than the crossover length $R_{\text{c}}\approx 1.1a{\left(R/a\right)}^{0.45}\ll R$ with R the radius of the unperturbed spherical vesicle. Vahid and Idema (2016) predicted that two identical conical inclusions would attract each other when placed at the same longitudinal coordinates on a membrane tubule, in contrast to the repulsion in the case of quasiplanar membranes. When the conical inclusions are situated on the same transversal coordinates, the elastic interaction induced by the tubular membrane is repulsive at small separations and attractive at large separations.

## 3 Numerical Studies

**FIGURE 3:**
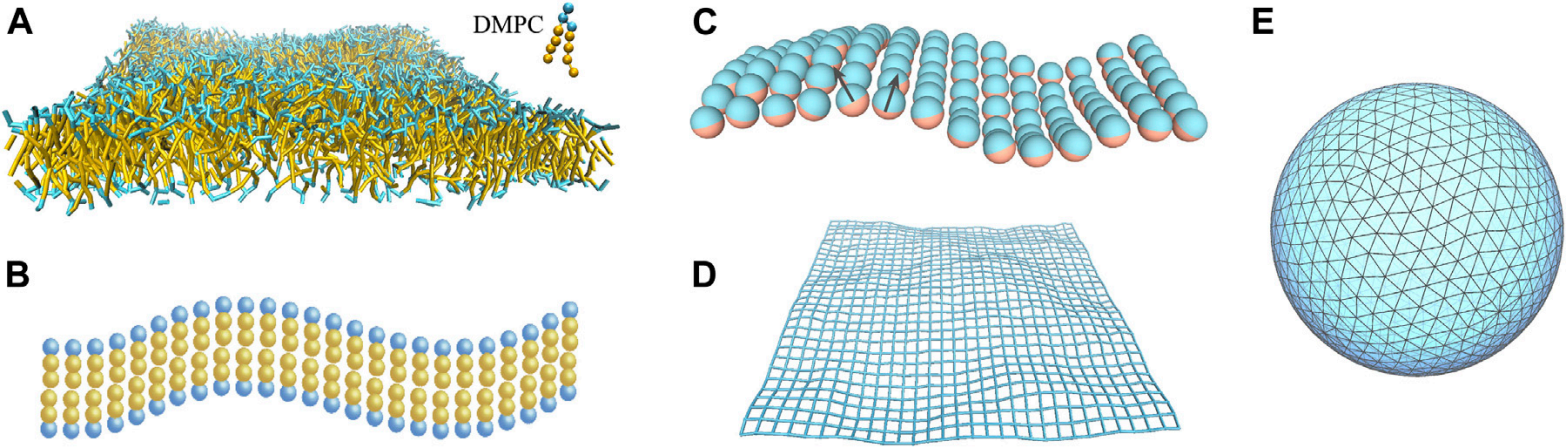
Computational models for fluid membranes at different levels of resolution. **(A)** Coarse-grained DMPC bilayer used in dissipative particle dynamics simulations. Water molecules are not shown here for clarity. **(B)** Cooke-Deserno model (Cooke and Deserno, 2005) with each lipid consisting of a hydrophilic head bead (in blue) and two hydrophobic tail beads (in yellow). Stable fluid bilayer can be simulated with a proper choice of tail-tail attraction without explicit solvent. **(C)** One-particle thick model (Yuan et al., 2010; Shiba and Noguchi, 2011). Any two particles experience short-range repulsion and long-range attraction. The particle-particle interaction depends on their orientations as specified by the normal vectors (black arrows). **(D)** Discretized elastic sheet model for a quasi-planar fluid membrane (Weikl, 2001; Dommersnes and Fournier, 2002). The membrane configuration is described by the displacement field *h*_*i*_ with respect to the horizontal reference plane at each discrete site *i*. **(E)** Triangulated surface model for vesicular membranes (Kroll and Gompper, 1992; Sunil Kumar et al., 2001; Hu et al., 2011). The angle between the normal vectors of each two neighboring triangles determines the local curvature of the membrane.

As stated above, theoretical studies of membrane-mediated interactions are often restricted to small deformations of the membranes and do not take into account specific lipid-protein interactions. Numerical simulations can overcome those limitations, e.g., specific lipid-protein interactions can be dealt with atomistic molecular dynamics simulations. Figure 3 shows a variety of fluid membrane models used in coarse-grained simulations.

### 3.1 Short-Range Interaction

Monte Carlo (MC) (Sintes and Baumgärtner, 1997; West et al., 2009) and molecular dynamics (MD) (Venturoli et al., 2005; de Meyer et al., 2008; Schmidt et al., 2008; West et al., 2009) simulations based on coarse-grained models of lipids and proteins have been used to investigate membrane-mediated short-range interactions between two cylindrical protein inclusions with emphasis on the generic feature of the interactions. Simulation study of proteins without hydrophobic mismatch was first done by Sintes and Baumgärtner (1997), who considered two rigid cylinders (of diameter two to four times of lipid width *σ*) embedded in a lipid bilayer whose bending deformation was strongly suppressed. They found a depletion-induced attraction for inclusion separation *R* < *σ* and an oscillating interaction for *σ* < *R* < 6*σ* attributed to inhomogeneous distribution and orientational fluctuations of lipid chains around inclusions. In simulation studies with hydrophobic mismatch between the bilayer and protein inclusions, the bilayer thickness profile around single inclusions was found to exhibit similar nonmonotonic behavior (Venturoli et al., 2005; de Meyer et al., 2008; Schmidt et al., 2008; West et al., 2009), consistent with the elastic theory. The potential of mean force (PMF) between two rigid inclusions depends on inclusion size (de Meyer et al., 2008) and lipid-protein interaction (West et al., 2009). For protein inclusions that have no or weak affinity to lipid chains, the PMF is attractive at smaller separations and repulsive at intermediate separations (de Meyer et al., 2008; Schmidt et al., 2008), also in accordance with the elastic theory. For protein inclusions strongly attracting lipid chains, the PMF is highly oscillatory with a repulsion at close separations (West et al., 2009). Protein inclusions with hydrophobic thickness larger than that of the bilayer can be tilted (Venturoli et al., 2005; Klingelhoefer et al., 2009) or even bent (Venturoli et al., 2005) in the membrane in order to avoid exposure of their hydrophobic domains.

All-atom simulations can provide atomistic details of the bilayer deformation around single protein inclusions. Kim et al. (2012) performed all-atom MD simulations to measure the thickness profile of different bilayers embedding a Gramicidin A channel, and found qualitative discrepancy from theoretical predictions, probably due to specific protein-lipid interactions that are not addressed in the simplified membrane elasticity model. Mondal et al. (2011) studied the energetics of lipid bilayer deformations around a noncylindrical protein inclusion like G-protein coupled receptors by taking the continuum theory where the membrane-protein boundary conditions were extracted from atomistic MD simulations. Argudo et al. (2017) revisited the atomistic MD simulations of a Gramicidin A channel embedded in a POPC bilayer and showed that the membrane deformations and tilt of the ionic channel can be quantitatively captured by a refined bilayer model that incorporates the chemistry and geometry of the protein inclusions. However, due to the computational cost, it remains challenging to measure the membrane-mediated short-range interactions between two protein inclusions from all-atom MD simulations.

### 3.2 Long-Range Interaction

To directly measure the fluctuation-induced interactions remains a computationally difficult task, since they are weak and often coupled with the curvature-induced elastic interactions. Weikl (2001) studied the fluctuation-induced aggregation of protein inclusions much more rigid than the fluid membrane *via* MC simulations, where the membrane was represented by a discretized 2D elastic sheet and protein inclusions occupy single vacant sites on the membrane surface. The systems with inclusions of *κ*_p_ and ${\bar{\kappa }}_{\text{p}}$ two order-of-magnitude larger than those of the membrane were found to separate into inclusion-rich and inclusion-poor phases even in the absence of any direct protein-protein attraction, whereas the systems with no contrast in the Gaussian moduli ( ${\bar{\kappa }}_{\text{p}}=\bar{\kappa }$ ) exhibit the same critical point as if the membrane were completely flat without shape fluctuations. This finding points out that a difference in Gaussian moduli is necessary for a fluctuation-induced interaction between membrane inclusions, as mentioned in Section 2.2.1. Pezeshkian et al. (2016) reported from MD simulations the clustering of rigid pentagon shaped nanoparticles, coarse-grained model of bacterial Shiga toxin, on lipid membranes driven by the fluctuation-induced attraction. Very recently, Sadeghi and Noé (2021) extracted from MD simulations the membrane-mediated effective interactions between protein particles embedded in a fluid membrane modeled by particle-based elastic sheet. The interaction varies non-monotonically with interparticle separation and has a depth of about *k*_B_ *T* for different values of protein stiffness, which can not be accounted for by the sum of the two-body interactions $F_{\text{fl}}+F_{\text{el}}\approx -6k_{\text{B}}T{\left(a/R\right)}^{4}+4\pi \kappa \left(\alpha_{1}^{2}+\alpha_{2}^{2}\right){\left(a/R\right)}^{4}$ . It is not clear whether the discrepancy is model specific.

**FIGURE 4:**
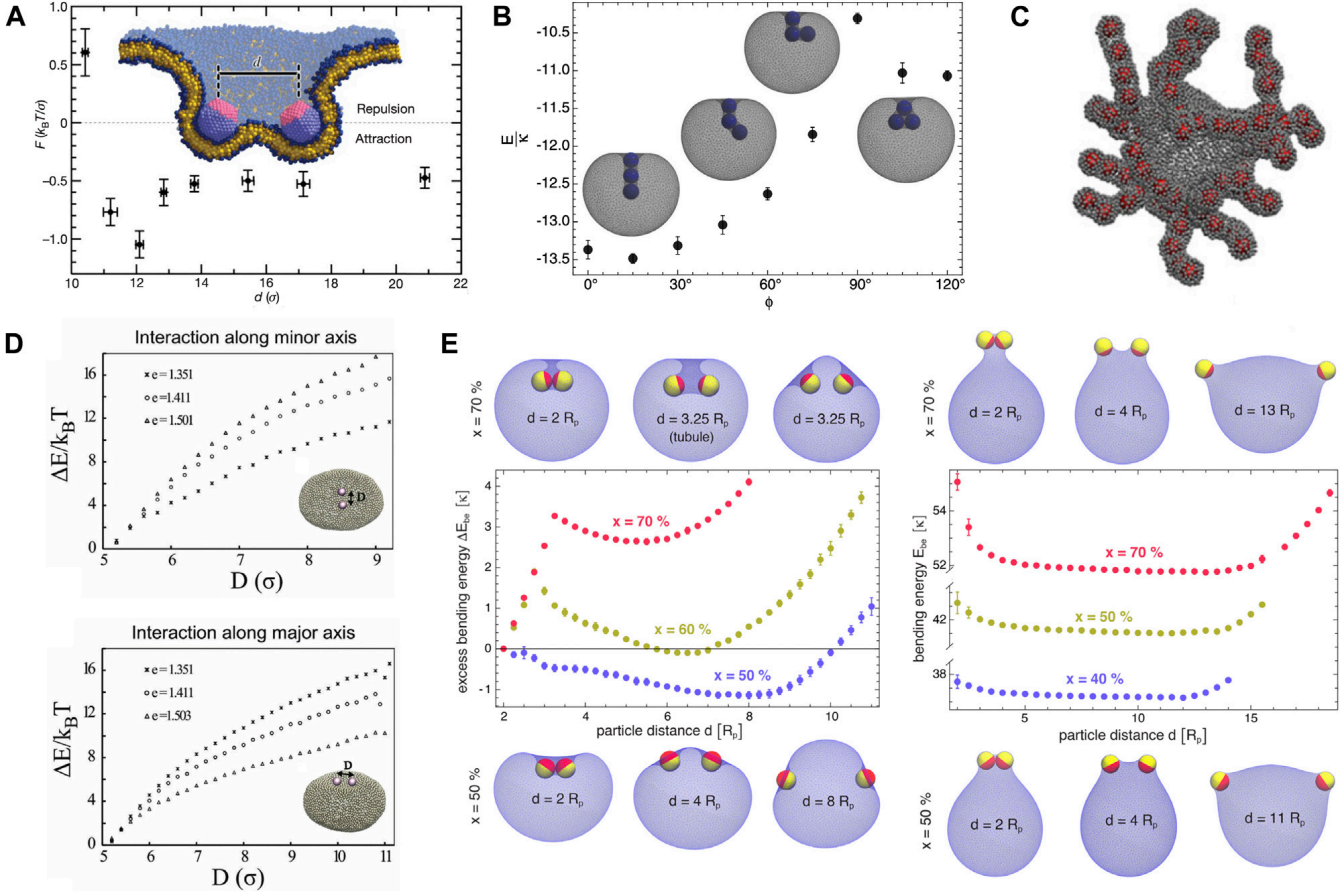
Membrane-mediated interactions between quasi-spherical or spherical particles adsorbed on fluid membranes obtained from coarse-grained MD or MC simulations. **(A)** Force *F* versus center-to-center distance *d* between two capsids (diameter of 8 nm) strongly bound to a fluid bilayer. Reprinted by permission from Springer Nature Customer Service Centre GmbH: Reynwar et al. (2007), Copyright (2007). The minimal attraction at *d* ≈ 12 *σ* = 12 nm is about *k*_*B*_ *T*/*σ* ∼ 4 pN. **(B)** Rescaled total energy *E*/*κ* of a vesicle with three adsorbed particles wrapped by membrane tubes as a function of the angle *ϕ* between the particles (Bahrami et al., 2012), copyright (2012) by the American Physical Society. Insets are energy-minimum configurations for *ϕ* = 0°, 45°, 90°, and 120°. The most stable configuration corresponds to linear aggregation of the three particles inside the membrane tube, reflecting membrane-mediated strong attraction between the particles. The vesicle membrane has a fixed area of A and a fixed enclosed volume of $V=0.88\times \left(4\pi /3\right){\left[A/\left(4\pi \right)\right]}^{3/2}$ . The particle-membrane adhesion energy per area is $U=2\kappa /R_{\text{p}}^{2}$ with *κ* membrane bending rigidity and *R*_p_ particle radius. **(C)** Snapshot of aggregates of spherical particles adsorbed to vesicular membranes from MC simulations at constant temperature (Šarić and Cacciuto, 2012b), copyright (2012) by the American Physical Society. **(D)** Interaction energy Δ*E* versus distance *D* of two spheres bound to an ellipsoidal vesicle with eccentricity *e* (Vahid et al., 2017), with permission of The Royal Society of Chemistry. **(E)** Excess bending energy Δ*E*_be_ of a vesicle versus distance *R* of two Janus spherical particles adhered to the outside (left panel) or inside (right panel) of the membrane, reprinted with permission from Bahrami and Weikl (2018), copyright 2018 American Chemical Society. The adhesive cap in yellow is fully covered by the membrane and has an area fraction of *x*. For *x* = 60*%* and 70%, the increase of Δ*E*_be_ at small values of *d* corresponds to curvature-induced elastic attraction between the particles. The decrease of Δ*E*_be_ at intermediate values of *d* corresponds to curvature-induced repulsion between the particles.

MD simulations with coarse-grained models of lipid membranes at different levels of resolution (Reynwar et al., 2007; Olinger et al., 2016; Xiong et al., 2017; Spangler et al., 2018; Noguchi, 2016; Noguchi and Fournier, 2017), MC simulations (Bahrami et al., 2012; Šarić and Cacciuto, 2012b,a; Vahid et al., 2017; Bahrami and Weikl, 2018; Bonazzi and Weikl, 2019) and numerical minimizations (Reynwar and Deserno, 2011; Schweitzer and Kozlov, 2015) based on mesoscopic elastic surface models showed that proteins or particles adhering to membranes experience curvature-induced interactions, which are strongly attractive in many cases and can drive particle assembly on the membranes. We first review the studies of spherical particles. Reynwar et al. (2007) computed directly from coarse-grained MD simulations the force between two capsids adhering strongly to a lipid bilayer, and obtained repulsive forces at small capsid separations followed by attractive ones at large separations as shown in Figure 4A, seemingly contradictory to the theoretical prediction of pure elastic repulsion for conical inclusions in Section 2.2.1. By using the Surface Evolver package (Brakke, 1992) to numerically minimize the energy of a membrane adhering to two spherical particles, Reynwar and Deserno (2011) confirmed later that the curvature-induced force is indeed repulsive for small contact angles (i.e., weak adhesion), whereas for contact angles larger than 90° (i.e., strong adhesion), the force changes from repulsive to attractive with increasing separation, consistent with their MD results in Ref (Reynwar et al., 2007).

Using a triangulated surface model for vesicle membranes, Bahrami et al. (2012) determined from simulated annealing MC simulations the minimum-energy shape of vesicle membranes interacting with adhesive spherical particles, and discovered stable membrane tubules that wrap one row of two or three particles; *see* Figure 4B. As shown in Figure 4C, similar membrane tubular structures were observed in constant-temperature MC simulations by Šarić and Cacciuto (2012b), who also reported linear aggregation of spherical particles adsorbed on vesicle membranes (Šarić and Cacciuto, 2012a). MD simulations (Reynwar et al., 2007; Xiong et al., 2017; Spangler et al., 2018) with molecular models for flat lipid bilayers showed that linear aggregation of adsorbed spherical particles induces membrane tubulation and vesiculation. These studies point towards curvature-induced strong attractions between spherical particles adhering to fluid membranes. Vahid et al. (2017) further demonstrated that, in the case of quasi-spherical vesicles, the curvature-induced attraction between two adsorbed spherical particles becomes weaker as the vesicle gets bigger. In the case of quasi-ellipsoidal vesicles, the attraction for two particles placed along the major axis is different from that along the minor axis, and their relative magnitude depends on the ellipticity of the vesicles; *see* Figure 4D. This MC simulation result suggests that the background curvature of closed membranes affects the curvature-mediated attraction. In the extension of their previous study (Bahrami et al., 2012), Bahrami and Weikl (2018) reported that two spherical Janus particles with one side strongly adhering to vesicle membranes can attract or repel each other, depending on the area fraction of the adhesive side and on the shape (i.e., concave or convex) of the adhering membrane segments; *see* Figure 4E. In all these simulated systems (Reynwar et al., 2007; Olinger et al., 2016; Xiong et al., 2017; Spangler et al., 2018; Noguchi, 2016; Noguchi and Fournier, 2017; Bahrami et al., 2012; Šarić and Cacciuto, 2012b,a; Vahid et al., 2017; Bahrami and Weikl, 2018; Bonazzi and Weikl, 2019; Reynwar and Deserno, 2011; Schweitzer and Kozlov, 2015), particle-membrane adhesion energy, membrane bending energy, and possible constraint due to conservation of the volume enclosed by membranes determine together the optimal membrane shape and thus how the curvature-induced interaction varies with inter-particle distance.

**FIGURE 5:**
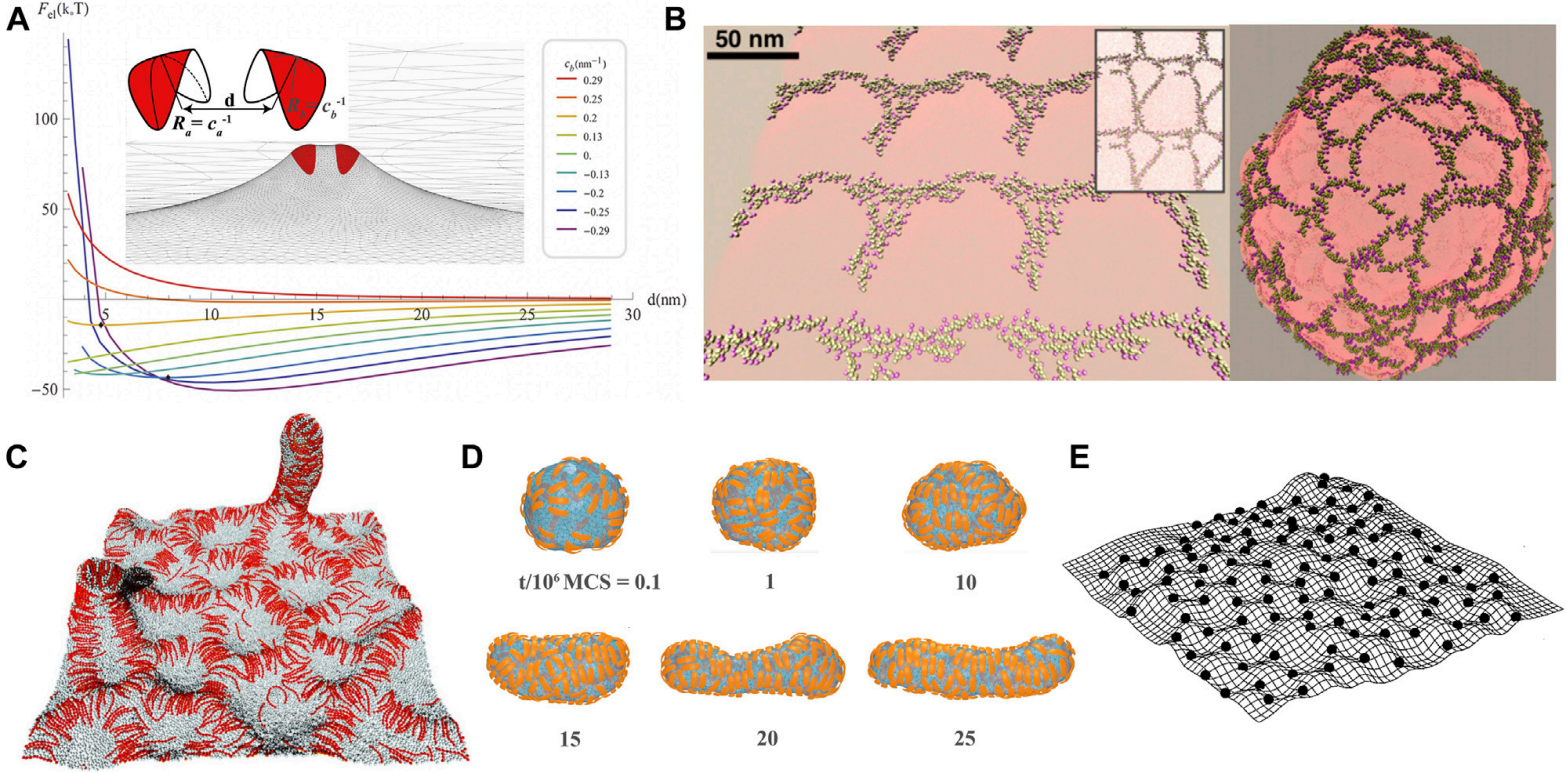
Membrane-mediated interactions between anisotropic protein inclusions **(A)** and assembly of anisotropic proteins on membranes **(B**–**E)**. **(A)** Curvature-induced elastic energy *F*_el_ versus distance *d* of two BAR-like membrane scaffolds for different values of scaffold curvature *c*_*b*_ (Schweitzer and Kozlov, 2015)(CC BY 4.0). The BAR-like scaffold has principle curvatures *c*_*a*_ and *c*_*b*_ as illustrated in the inset. When projected to initial membrane plane, the scaffold has an elliptical shape with semi-axes of 6.5 and 1.5 nm. The membrane bending rigidity *κ* = 20 *k*_B_ *T*. **(B)** Linear aggregation of N-BARs on flat and vesicular membranes observed in coarse-grained MD simulations (Simunovic et al., 2013). Copyright (2013) National Academy of Sciences. **(C)** Side-by-side alignment of BAR-like particles on membrane bulges and tubes (Noguchi, 2016; Noguchi and Fournier, 2017), with permission of The Royal Society of Chemistry. **(D)** MC sequence of configurations of an initially spherical vesicle remodeled by arc-shaped scaffold. Reprinted from Bonazzi and Weikl (2019), copyright (2019), with permission from Elsevier. Free scaffolds unbound to the membrane are not shown here for clarity. **(E)** Assembly of saddle-shaped protein inclusions (modeled by point-like particles that locally impose two opposite eigenvalues of the membrane’s curvature tensor) into regular pattern on the membrane. Reprinted from Dommersnes and Fournier (2002), copyright (2002), with permission from Elsevier.

Numerical studies of anisotropic protein inclusions or scaffolds interacting with fluid membranes have also revealed that the membrane-mediated attractions are important for the protein to assemble and to remodel the membranes. Using Surface Evolver to find the minimum-energy shape of membranes interacting with two rigid protein scaffolds, Schweitzer and Kozlov (2015) showed that for circular scaffolds with anisotropic curvature (saddle-like or ellipsoidal shape), or isotropically curved scaffolds of elongated shapes (noncircular footprint on the membrane), the curvature-induced interaction is repulsive at small inter-scaffold separations and attractive at large separations. Specifically, the curvature-mediated attraction between two BAR-domain-like scaffolds was found to be very strong; *see* Figure 5A. Using a coarse-grained molecular model of N-BAR domain, Simunovic et al. (2013) showed from MD simulations that the proteins assemble on flat or vesicle membranes at low concentrations and form a mesh of linear aggregates as shown in Figure 5B. Noguchi (2016), and Noguchi and Fournier (2017) studied the assembly of arc-shaped proteins on flat membranes with coarse-grained MD simulations, and found side-by-side alignment of the proteins around membrane tubules; *see* Figure 5C. The membrane-mediated side-by-side arrangement was also reported by Bonazzi and Weikl (2019) in MC simulations of arc-shaped particles remodeling an initially spherical vesicle modeled by a triangulated surface; *see* Figure 5D, where the rather loose arrangement of particles has been experimentally reported for N-BAR proteins interacting with membrane tubules (Daum et al., 2016). We note that, in addition to the membrane-mediated indirect interactions, direct protein-protein interactions may also play a part in the assembly of anisotropic proteins on membranes. For instance, the helical arrangement of N-BAR proteins around membrane nanotubes found in coarse-grained MD simulations (Simunovic et al., 2016) is very likely due to the direct attraction between the proteins. Using the same discrete model as in Ref (Weikl, 2001) and treating saddle-like inclusions as point-like constraints that impinge anisotropic curvature on the membrane, Dommersnes and Fournier (2002) simulated the assembly of those inclusions, assisted by the curvature-induced attraction, into regular arrays that shape the membrane into the experimentally observed egg-carton pattern as shown in Figure 5E.

It is worthy to mention the membrane-mediated interactions in the systems of cell adhesion that is mediated by the specific binding of membrane-anchored receptors and ligands. The receptor-ligand complexes constrain the local separations of the two adhering membranes and thus experience fluctuation-induced attractions (Bruinsma and Pincus, 1996; Krobath et al., 2009; Weikl, 2018). An important biological consequence of these membrane-mediated attractions is the cooperative binding of cell adhesion proteins, as corroborated by MD simulations (Hu et al., 2013; Hu et al., 2015; Xu et al., 2015) and experiments (Steinkühler et al., 2018).

## 4 Summary and Outlook

We have reviewed both theoretical and numerical studies on membrane-mediated interactions between protein inclusions. The continuum theories for the short- and long-range interactions are based on membrane elasticity models at different length scales. A natural question to ask is whether these theories can be cast into a unified framework. Agrawal et al. (2016) introduced in the Helfrich Hamiltonian additional terms for the jump of displacement and rotation angles that account for the hydrophobic mismatch and structural rearrangement of lipids around the protein inclusions, and showed that the curvature-induced repulsion between conical protein inclusions can be reduced by orders of magnitude. This prediction shall be tested by large scale simulations with coarse-grained molecular models or even atomistic models for proteins and lipid bilayers.

Despite the physical insights provided by numerous simulation studies, quantitative comparison between simulations and existing theories is still very limited. Such comparison would be invaluable for checking the validity of the assumptions involved in the theories. Moreover, simulations on membrane remodeling by isotropic or anisotropic proteins or particles stimulate the necessity of developing theories for membrane-mediated interactions between protein inclusions under large membrane deformations. Theoretical approach based on line integral of stress tensor in Refs (Müller et al., 2005, 2007) represents a possible choice.

Finally, we would like to point out that the real cell environment is much more complicated than the model systems considered in the theories and simulations. Cell membranes are linked to cytoskeleton that undergoes active deformations. The membrane proteins or nanoparticles adhering to the membranes may also be associated with active processes (Ramaswamy et al., 2000). It is interesting to ask how such nonequilibrium factors contribute to or even change the membrane-mediated interactions between protein inclusions.
